# Translation of paclitaxel-induced peripheral neurotoxicity from mice to patients: the importance of model selection

**DOI:** 10.1097/j.pain.0000000000003268

**Published:** 2024-05-02

**Authors:** Guido Cavaletti, Paola Alberti, Annalisa Canta, Valentina Carozzi, Laura Cherchi, Alessia Chiorazzi, Luca Crippa, Paola Marmiroli, Cristina Meregalli, Eleonora Pozzi, Virginia Rodriguez-Menendez, Christian Steinkühler, Simonetta Andrea Licandro

**Affiliations:** a Experimental Neurology Unit, University of Milano-Bicocca, Monza, Italy; b Fondazione IRCCS San Gerardo dei Tintori, Monza, Italy; c New Drug Incubator Division, Italfarmaco S.p.A., Milan, Italy

**Keywords:** Paclitaxel, Mice, Animal models, Pain

## Abstract

Paclitaxel-induced peripheral neurotoxicity (PIPN) is a potentially dose-limiting side effect in anticancer chemotherapy. Several animal models of PIPN exist, but their results are sometimes difficult to be translated into the clinical setting. We compared 2 widely used PIPN models characterized by marked differences in their methodologies. Female C57BL/6JOlaHsd mice were used, and they received only paclitaxel vehicle (n = 38) or paclitaxel via intravenous injection (n = 19, 70 mg/kg) once a week for 4 weeks (Study 1) or intraperitoneally (n = 19, 10 mg/kg) every 2 days for 7 times (Study 2). At the end of treatment and in the follow-up, mice underwent behavioral and neurophysiological assessments of PIPN. At the same time points, some mice were killed and dorsal root ganglia, skin, and sciatic and caudal nerve samples underwent pathological examination. Serum neurofilament light levels were also measured. The differences in the neurotoxicity parameters were analyzed using a nonparametric Mann-Whitney test, with significance level set at *P* < 0.05. Study 1 showed significant and consistent behavioral, neurophysiological, pathological, and serological changes induced by paclitaxel administration at the end of treatment, and most of these changes were still evident in the follow-up period. By contrast, study 2 evidenced only a transient small fiber neuropathy, associated with neuropathic pain. Our comparative study clearly distinguished a PIPN model recapitulating all the clinical features of the human condition and a model showing only small fiber neuropathy with neuropathic pain induced by paclitaxel.

## Abstract 2

Pain is only one symptom of chemotherapy-induced peripheral neurotoxicity, so very careful selection of the animal models is required for reliable translation to the bedside.

## 1. Introduction

Chemotherapy-induced peripheral neurotoxicity (CIPN) represents, after hematological toxicities, the main cause of reduction or suspension of some antineoplastic treatments.^3,6,19^ All cancers with high incidence in Western countries (ie, breast, lung, gastrointestinal tract, and prostate) are candidates for chemotherapy treatment with neurotoxic drugs, alone or in combination. Remarkably, the incidence of CIPN can exceed 80% of treated patients, and it may be irreversible, with severe effects on their quality of life.^6,33,39,41^ Since no neuroprotective treatments are available to prevent CIPN, modification of the planned chemotherapy regimen is required to preserve the functional integrity of the peripheral nervous system of the treated subject, but this can negatively affect the oncological outcome.

The mechanisms leading to CIPN are still not completely understood.^7,40^ Based on preclinical evidence, oxidative stress, interference with tubulin with damage to the cytoskeleton and impairment in axonal transport, neuronal drug accumulation due to the activity of specific transporters, and neuroinflammation have been suggested as possible pathogenic events.^4,17,18,35,36,38^

Despite intense investigation using in vitro cellular systems, the use of animal models remains essential in experimental CIPN.^44^ However, some of these models reproduce only partially the clinical picture of the tested drugs, thus raising concern when the preclinical results are translated into clinical practice.^16^

Paclitaxel (PTX) is particularly relevant among the neurotoxic chemotherapy drugs since it is very widely used and effective, allowing extremely long disease-free survival (particularly in patients with breast cancer).^15,34^ Modeling PTX-induced peripheral neurotoxicity (PIPN) is particularly challenging because the drug has a complex neurotoxicity profile, characterized by acute painful syndrome ensuing in most patients after each PTX administration and remitting before the subsequent cycle, followed by a much more severe chronic sensorimotor polyneuropathy.^2,14,20,23,40,42^

Several rodent models have been proposed to reproduce PIPN,^29^ but their real capacity to translate all the clinical features to the animal setting remains unsettled. The main differences existing among these models regard the route of delivery, the single and total dose intensities, the duration of treatment, and the interval between administrations. To clarify the capacity of these models to reliably mimic the features of human PIPN, we compared, using an extensive multimodal approach, 2 well-characterized PIPN models developed in mice presenting remarkable differences in their experimental design and outcomes.

Our comparison evidenced that only one of the experimental paradigms is able to recapitulate all the features of human PIPN, thus further highlighting the need for a very careful selection of the preclinical model to obtain reliable results to be translated into the clinical setting.

## 2. Materials and methods

### 2.1. Animals and housing conditions

Animals underwent health evaluation shortly after arrival. Their care and husbandry were in compliance with national (D.L.vo n. 26/2014) and international laws and policies (EEC Council Directive 86/609, OJ L 358, 1, Dec 12, 1987; Guide for the Care and Use of Laboratory Animals, US National Research Council, 2011). Animals were housed under controlled conditions (room temperature: 22 ± 2°C, room relative humidity: 55 ± 10%, 24-hour cycle of 12 hours light/12 hours dark, 7 am–7 pm). The study protocol was submitted to and approved by the University of Milano-Bicocca Animal Welfare Board and the Italian Ministry of Health (approval number 777/2022-PR, Dec 22, 2022).

**Figure 1.**
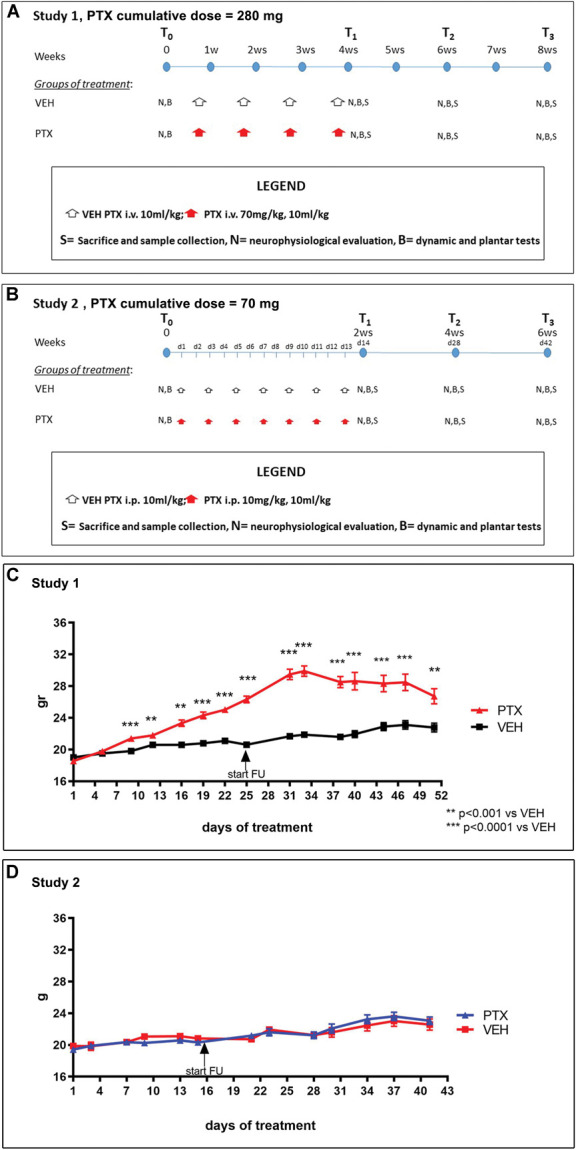
Flowchart of the animal studies (A = study 1, B = study 2) and changes in animals’ body weight along the studies (C = study 1, D = study 2).

For the experiment, a total of 76 female C57BL/6JOlaHsd mice (Envigo, Bresso, Italy) aged 8 weeks on arrival were used. Figures 1A and B present the flowcharts of study 1 and study 2, respectively.

#### 2.1.1. Study 1

After randomization based on the nerve conduction studies and behavioral tests (see below) performed to ensure comparable baseline values, 19 mice were used as control and received only PTX vehicle (VEH) while 19 mice were PTX treated. Paclitaxel (LC Laboratories, Woburn, MA) was administered by intravenous (i.v.) injection once a week for 4 weeks (70 mg/kg, volume of administration = 10 mL/kg, used immediately after dilution with 5% tween 80, 5% ethanol 100, 90% saline solution, cumulative dose = 280 mg/kg); after the treatment, mice were left untreated for a 4-week follow-up period.^9^

#### 2.1.2. Study 2

After randomization performed as in study 1, 19 mice were used as control and received only PTX VEH while 19 mice were PTX treated. Paclitaxel was administered intraperitoneally (i.p.) every 2 days 7 times (10 mg/kg, volume of administration = 10 mL/kg, used immediately after dilution with 10% Cremophor El, 10% ethanol 100 (1:1) in 80% saline solution, cumulative dose = 70 mg/kg); after the treatment, mice were left untreated for a 4-week follow-up period.^21^

### 2.2. Neurotoxicity assessment

#### 2.2.1. Noninvasive tests

Noninvasive tests were performed in both studies on all the animals at baseline, after the end of treatment, and after 2 and 4 weeks of follow-up. Whenever possible, data for all investigations were recorded online using software packages specifically designed for the purposes of the test facility. When online recording was not possible, handwritten raw data sheets were used. The data were subsequently entered manually into the computer, and the raw data sheets were archived (available at https://doi.org/10.17632/f8gvnh6d63.1). All behavioral tests were performed by a single blinded examiner.

##### 2.2.1.1. Nerve conduction studies

The development of PIPN was assessed by evaluating the sensory nerve conduction velocity (SNCV) and sensory nerve action potential amplitude (SNAP) of caudal and digital nerves, using an electromyography apparatus (Matrix Light, Micromed, Treviso, Italy). Sensory nerve conduction velocity and SNAP were measured by placing a couple of needles recording electrodes (cathode and anode) at the base of the tail (for caudal nerve recordings) or at the ankle bone (for digital nerve recordings) and a couple of stimulating electrodes 3.5 cm far from the recording points (for caudal nerve recordings) or close to the fourth toe (for digital nerve recordings). Latencies were measured from stimulus onset, and peak-to-peak amplitudes were calculated. The SNCV was calculated considering the distance between the recording and stimulating points divided by the latency from the stimulus artifact to the onset of the first peak of the elicited action potential. The intensity, duration, and frequency of stimulation were set up to obtain optimal results and the maximal amplitude of the SNAP. All the neurophysiological determinations were performed with the animals under isoflurane anesthesia along the whole procedure with continuous monitoring of vital signs and body temperature.^5,27^

##### 2.2.1.2. Dynamic Aesthesiometer Test

The mechanical nociceptive threshold was assessed using a Dynamic Aesthesiometer Test (model 37450, Ugo Basile Biological Instruments, Comerio, Italy), which generated a linearly increasing mechanical force. At each time point, after the acclimatization period, a servo-controlled mechanical stimulus (a pointed metallic filament, 0.5-mm diameter) was applied to the plantar surface of the hind paw, which exerted a progressively increasing punctuate pressure, reaching up to 15 g within 15 seconds. The pressure evoking a clear voluntary hind-paw withdrawal response was recorded automatically and taken as the mechanical nociceptive threshold. The mechanical threshold was always assessed on alternative sides every 2 minutes on 3 occasions to yield a mean value. The results represented the maximal pressure (expressed in grams) tolerated by the animals. There was an upper limit cutoff of 20 seconds, after which the mechanical stimulus was automatically terminated.^22^

##### 2.2.1.3. Plantar Test

The withdrawal latency to an infrared-generated heat stimulus was determined using a Plantar Test apparatus (Ugo Basile Biological Instruments). The animals were placed in a transparent plastic cage on an elevated plexiglass mesh table; after habituation, a movable infrared radiant heat source (IR 40 mW/cm^2^) was placed directly under the plantar surface of the hind paw, and the time to hind-paw withdrawal was monitored (withdrawal latency). The mean of 4 repeated trials was used for data analysis. There was an upper limit cutoff of 20 seconds.^10^

#### 2.2.2. Sampling and processing of organs

At the end of the treatment and after 2 and 4 weeks of follow-up, some of the animals were killed and blood and organs were collected. In particular, at each time point, sciatic nerves, proximal (1 cm from the base of the tail) and distal (5 mm from the distal end of the proximal segment) portions of the caudal nerves, and L4-L5 dorsal root ganglia (DRG) were collected from 5 animals/group for pathologic analysis while skin biopsies were obtained for morphological and morphometric examination.

##### 2.2.2.1. Neuropathology

Sciatic and caudal nerves and L4-L5 DRG were collected for morphological analysis and processed as previously described.^26^ Briefly, 1-µm–thick semithin sections of nerves and DRG were prepared from 3 animals/group, stained with methylene blue and examined with a Nexcope Ne920 AUTO light microscope (TiEsseLab Srl, Milano, Italy).

##### 2.2.2.2. Dorsal root ganglia morphometry

Dorsal root ganglia neurons from control mice and from mice treated with PTX collected at the end of the treatment period were used for the morphometric examination. Methylene blue–stained 1-μm–thick semithin sections were prepared and analyzed with a computer-assisted image analyzer using the ImageJ NIH software for DRG neuron analysis. The somatic, nuclear, and nucleolar size of DRG was measured in randomly selected sections according to previously reported methods on at least 300 DRG/mice.^11^

##### 2.2.2.3. Intraepidermal nerve fiber density assessment

To evaluate the intraepidermal nerve fiber (IENF) density, glabrous skin punches from the plantar hind paw were fixed in 2% paraformaldehyde lysine and periodate sodium, cryoprotected, and serially cut in 20-μm–thick sections. Three sections/animal were immunostained with rabbit polyclonal anti-protein gene product 9.5 (Proteintech, Illinois, Rosemont, IL) using a free-floating protocol. The total number of protein gene product 9.5–positive IENFs crossing the dermal-epidermal junction was counted under a light microscope at 40x magnification (Nexcope Ne920 AUTO light microscope, TiEsseLab Srl) by the same blinded examiner. Intraepidermal nerve fiber density was expressed as the number of IENFs/length of epidermis (mm).^8^

#### 2.2.3. Neurofilament light chain level analysis

Blood was obtained for neurofilament light chain (NfL) level measurement from 5 animals/group at the end of treatment and after 2 weeks of follow-up and from all the remaining animals after 4 weeks of follow-up. Serum was obtained by centrifugation of the clotted blood sample, and NfL concentration was measured using a Simoa NfL assay (Simoa NF-lightTM Advantage Kit [SR-X]) on an HD-1 Analyzer (Quanterix, Billerica, MA). The analyses of all the samples were conducted on one occasion, using one batch of reagents. Intra-assay coefficients of variation for quality control samples with NfL concentrations of 22.6 pg/mL and 50.2 pg/mL were below 10%.^24^

#### 2.2.4. Statistical evaluation

The numerosity of each group was defined using a power calculation based on the changes in nerve conductions study results, as previously done in similar experiments, and group randomization was based on baseline behavioral and neurophysiological results.^5,27^ Statistical analyses were performed using GraphPad Prism4 statistical package (GraphPad Software, San Diego, CA). The differences in body weight, nerve conduction studies, DRG neuron morphometric parameters, behavioral tests, NfL levels, and IENF densities were statistically analyzed using a nonparametric Mann-Whitney test, with significance level set at *P* < 0.05.

## 3. Results

### 3.1. General toxicity

#### 3.1.1. Study 1

The administration of PTX was tolerated by the animals, and no changes in their spontaneous behavior (eg, grooming, eating, making nests) were observed. However, a statistically significant increase in weight gain was recorded in PTX-treated vs VEH mice from the second week of PTX administration (Fig. 1C). At sacrifice, the PTX-treated animals showed subcutaneous fat accumulation, but after treatment withdrawal, this tendency to fat accumulation disappeared and the slope of the weight gain curve approached that observed in the VEH group.

#### 3.1.2. Study 2

The administration of PTX was well tolerated by the animals, and also in this cohort, no changes in spontaneous behavior of the treated animals were observed. After the fifth administration, one PTX-treated animal died. Necropsy did not provide any evidence explaining the death of this animal. The same body weight gain was observed throughout the experimental period in both groups (Fig. 1D).

### 3.2. Nerve conduction studies

**Figure 2.**
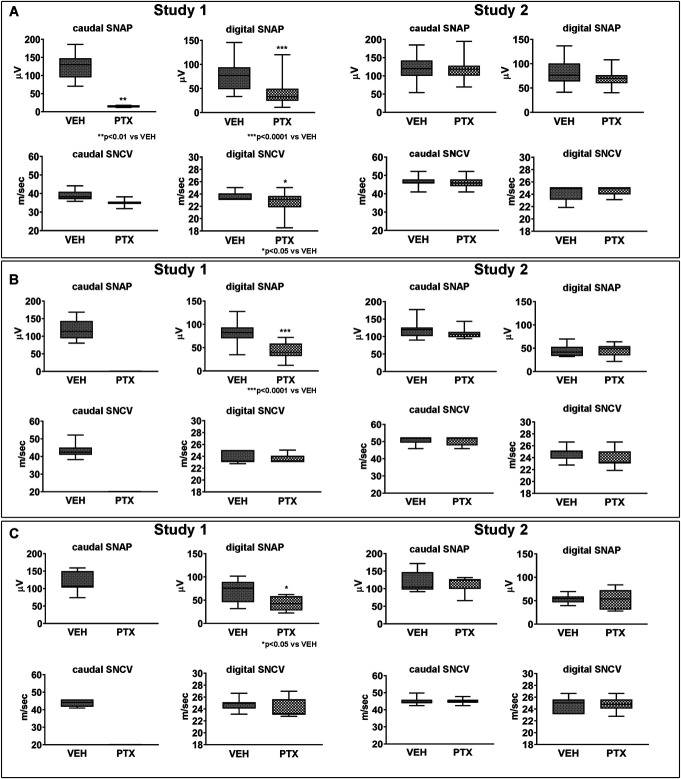
Neurophysiological results obtained in studies 1 and 2. In study 1, paclitaxel (PTX) administration induced significant changes in most sensory parameters vs control mice (VEH) in both caudal and digital nerves immediately after treatment (A), and these changes were partially present also after 2 (B) and 4 (C) weeks of follow-up. In study 2, PTX administration did not induce any significant change in sensory parameters vs VEH in both caudal and digital nerves immediately after treatment (A) and after 2 (B) and 4 (C) weeks of follow-up. SNAP, sensory nerve action potential amplitude; SNCV, sensory nerve conduction velocity; VEH, vehicle.

The neurophysiological results obtained at the end of treatment and during the follow-up are reported in Figure 2.

#### 3.2.1. Study 1

Caudal and digital SNAP and SNCV data obtained at baseline showed no differences between the control and treated groups (data not shown). At the end of treatment (Fig. 2A), statistically significant reductions in caudal (*P* < 0.01) and in digital SNAP (*P* < 0.0001) and in digital SNCV (*P* < 0.05) were observed in the PTX vs VEH group. After 2 (Figs. 2B) and 4 (Fig. 2C) weeks of follow-up, caudal SNAP and sensory SNCV were not recordable due to the severity of axonopathy. During the follow-up period, no statistically significant difference was observed between groups in digital SNCV after 2 weeks or after 4 weeks from treatment withdrawal while a significant reduction in digital SNAP (*P* < 0.0001 and *P* < 0.05 after 2 and 4 weeks of follow-up, respectively) was maintained at both time points.

#### 3.2.2. Study 2

Caudal and digital SNAP and SNCV data obtained at baseline showed no differences between the control and treated groups (data not shown). No significant differences in caudal or digital nerve conduction study results were observed after treatment or in the follow-up period (Figs. 2A–C).

### 3.3. Behavioral tests

**Figure 3.**
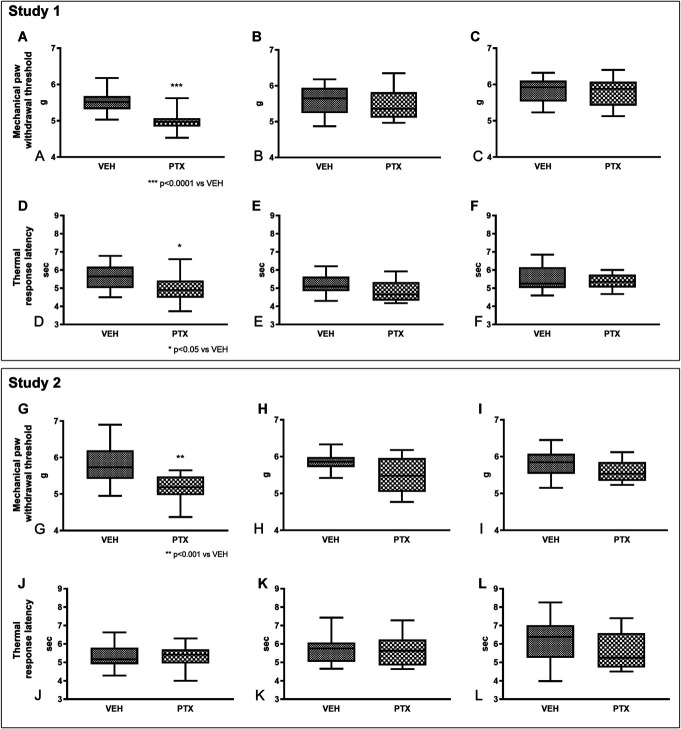
Behavioral changes induced by paclitaxel (PTX) administration in comparison with the results obtained in control (VEH) mice. In study 1, Dynamic Test results evidenced a significant, but reversible, reduction in mechanical threshold in PTX-treated mice (A = after treatment, B = after 2 weeks of follow-up, C = after 4 weeks of follow-up). The Plantar Test showed a similar, significant, and reversible reduction in thermal threshold (D = after treatment, E = after 2 weeks of follow-up, F = after 4 weeks of follow-up). By contrast, in study 2 only a significant reduction in the mechanical threshold after treatment was observed (G), while the results of all the remaining assessments were not significantly different from VEH mice (Dynamic Test G = after treatment, H = after 2 weeks of follow-up, I = after 4 weeks of follow-up; Plantar Test J = after treatment, K = after 2 weeks of follow-up, L = after 4 weeks of follow-up). VEH, vehicle.

At baseline, there was no statistically significant difference between groups in behavioral tests in both studies (data not shown). The results of the Dynamic Aesthesiometer Test for mechanical threshold and of the Plantar Test for thermal threshold obtained at the end of treatment and in the follow-up period are reported in Figure 3.

#### 3.3.1. Study 1

At the end of treatment (Fig. 3A), PTX induced the development of mechanical allodynia (*P* < 0.0001), but this alteration did not persist during (Fig. 3B) and at the end (Fig. 3C) of the follow-up period.

Similarly, at the end of treatment (Fig. 3D), PTX induced the onset of thermal hyperalgesia (*P* < 0.05), and also in this case, this alteration was no longer present during (Fig. 3E) and at the end (Fig. 3F) of the follow-up period.

#### 3.3.2. Study 2

At the end of treatment (Fig. 3G), PTX induced the development of mechanical allodynia (*P* < 0.001), but similarly to study 1, this alteration did not persist during the follow-up period (Figs. 3H and I).

In contrast to study 1, no alterations were observed at the end of treatment (Fig. 3J) and during the follow-up period (Figs. 3K and L) in thermal threshold.

### 3.4. Neuropathology

#### 3.4.1. Study 1

At the end of treatment and after 2 and 4 weeks of follow-up, no morphological alterations were observed in the DRG neurons or satellite cells belonging to both PTX and VEH groups.

**Figure 4.**
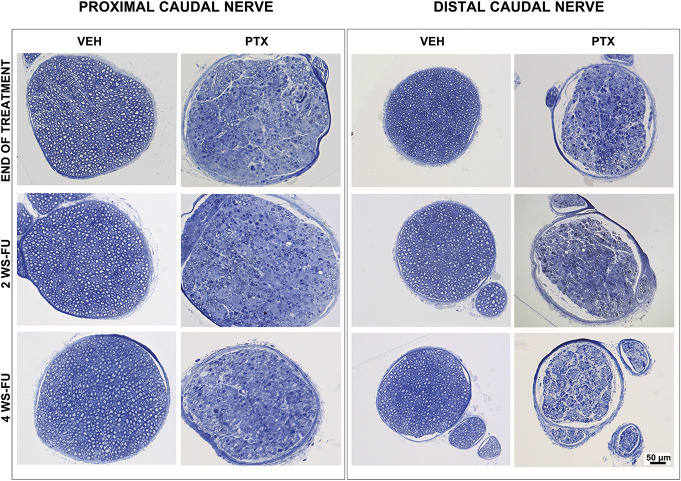
Representative light micrographs of the caudal nerves in study 1. Images are taken from control (VEH) and paclitaxel (PTX)-treated animals after treatment, after 2 (2 WS-FU) and 4 (4 WS-FU) weeks of follow-up. In the proximal segment, severe myelinated fiber loss due to axonopathy is induced by PTX administration at all the examined time points. In the distal segment, nearly complete myelinated fiber loss due to axonopathy is induced by PTX administration and is clearly evident at all the examined time points (1-μm–thick semithin sections, resin embedded, methylene blue staining). VEH, vehicle.

At the same time points, peripheral nerve samples were collected from the same animals. At the end of the treatment, the morphological analysis of the sciatic nerves revealed mild axonopathy with a few degenerated fibers in animals treated with PTX. At the end of the follow-up period, nearly complete recovery of this mild axonopathy was observed. By contrast, the morphological analysis of the proximal portion of the caudal nerve revealed severe axonopathy, with several degenerated fibers in the animals treated with PTX. In the follow-up period, only minimal signs of regeneration were evident, represented by small clusters of tiny myelinated fibers. The morphological analysis of the distal portion of the caudal nerves performed at the same time points revealed an even more severe axonopathy, with nearly complete loss of myelinated fibers. Representative proximal and distal caudal nerve images taken from samples obtained after treatment and at different time points in the follow-up are presented in Figure 4.

#### 3.4.2. Study 2

No pathological changes were observed in the DRG belonging to both PTX and VEH groups collected at the end of the treatment or in the follow-up period.

**Figure 5.**
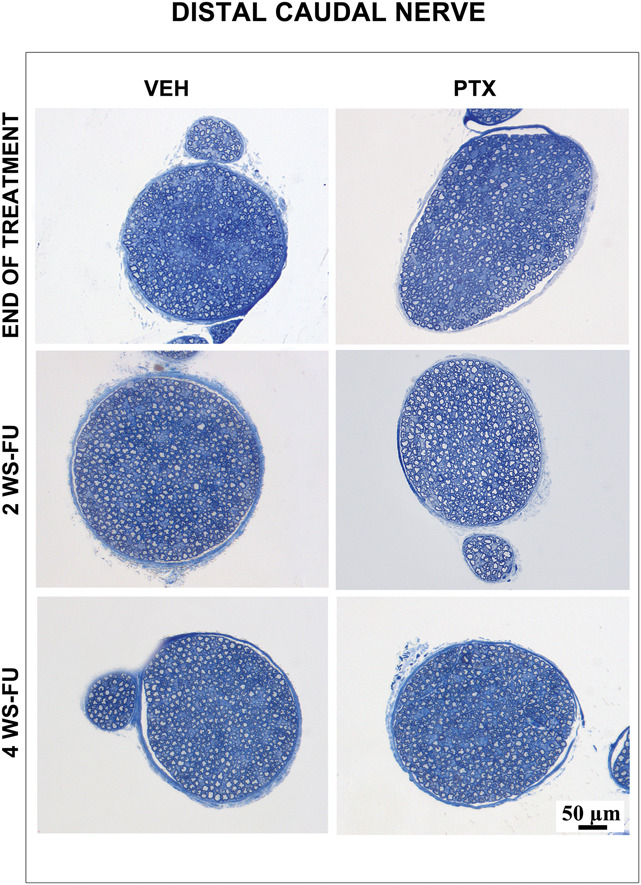
Representative light micrographs of the distal caudal nerves in study 2. Images are taken from control (VEH) and paclitaxel (PTX)-treated animals after treatment, after 2 (2 WS-FU) and 4 (4 WS-FU) weeks of follow-up. No pathological changes were observed in PTX-treated mice vs VEH animals at any of the examined time points (1-μm–thick semithin sections, resin embedded, methylene blue staining). VEH, vehicle.

No pathological changes were detected in all the nerve samples collected and examined at the same time point. Representative distal caudal nerve images taken from PTX and VEH animals are presented in Figure 5.

### 3.5. Dorsal root ganglia morphometry

#### 3.5.1. Study 1

The morphometric analysis performed on DRG neurons belonging to VEH or PTX-treated mice did not evidence any significant difference in somatic, nuclear, or nucleolar size (mean somatic size expressed in μ^2^ ± SD: VEH = 662.5 ± 304.9, PTX = 671.3 ± 320.3; mean nuclear size in μ^2^ ± SD: VEH = 110.2 ± 41.3, PTX = 108.6 ± 37.4; mean nucleolar size in μ^2^ ± SD: VEH = 10.4 ± 3.9, PTX = 11.4 ± 4.3).

#### 3.5.2. Study 2

Accordingly, also in study 2, no significant difference was observed in VEH vs PTX-treated mice in any of the investigated DRG neuron parameters (mean somatic size in μ^2^ ± SD: VEH = 573.2 ± 329.9, PTX = 567.6 ± 307.4; mean nuclear size in μ^2^ ± SD: VEH = 105.2 ± 42.1, PTX = 101.9 ± 39.8; mean nucleolar size in μ^2^ ± SD: VEH = 8.7 ± 3.1, PTX = 8.5 ± 3.2).

### 3.6. Intraepidermal nerve fiber density evaluation

**Figure 6.**
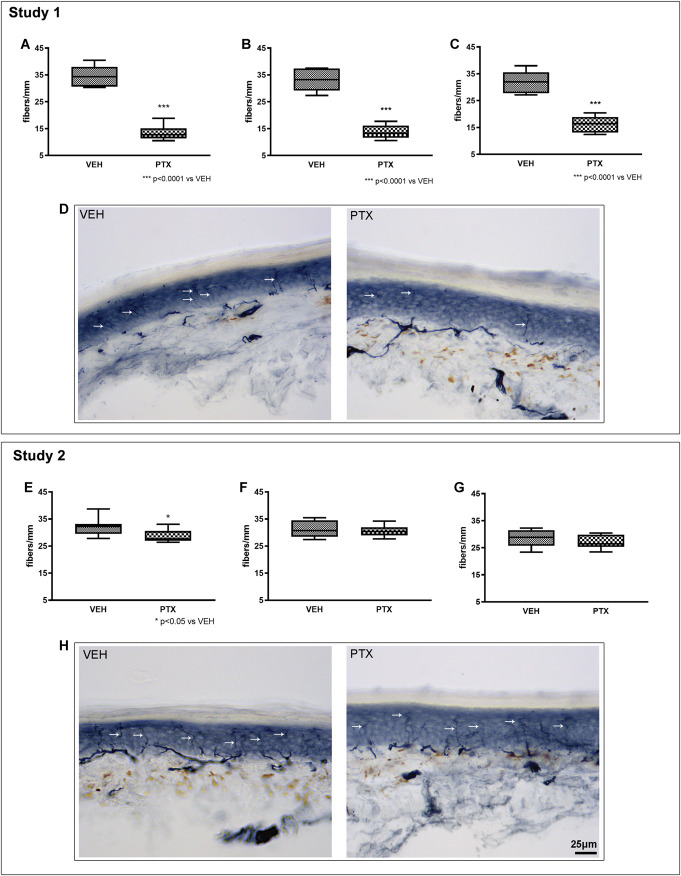
Comparison of intraepidermal nerve fibers (IENF) density. The assessments performed in study 1 (A = after treatment, B = after 2 weeks of follow-up, C = after 4 weeks of follow up) and in study 2 (E = after treatment, F = after 2 weeks of follow-up, G = after 4 weeks of follow-up) are reported. Paclitaxel (PTX) administration induced a significant reduction in IENF density vs controls (VEH) that was evident at each of the examined time points in study 1, while a significant reduction in IENF density was observed only after treatment in study 2, and its magnitude was remarkably lower if compared with that observed at the same time point in study 1. Panels (D and H) show representative images of skin biopsies obtained in study 1 and study 2, respectively. Arrows indicate the IENF (protein gene product 9.5 [PGP 9.5] immunostaining followed by HRP). VEH, vehicle.

At the end of treatment and after 2 and 4 weeks of follow-up, IENF density was assessed, and the results are represented in Figure 6.

#### 3.6.1. Study 1

At the end of treatment (Fig. 6A), a statistically significant decrease in IENF density was observed in the PTX vs VEH group (*P* < 0.0001), and this decrease persisted after 2 weeks of follow-up (Fig. 6B) and at the end of the follow-up period (Fig. 6C) (*P* < 0.0001 at both time points). Figure 6D shows the remarkable and significant decrease in IENF density induced by PTX administration.

#### 3.6.2. Study 2

At the end of treatment (Fig. 6E), a statistically significant decrease in IENF was observed in the PTX group (*P* < 0.5 vs VEH), but this decrease was no longer evident after 2 weeks of follow-up (Fig. 6F) and at the end of the follow-up period (Fig. 6G). Figure 6H shows the decrease in IENF density induced by PTX administration, clearly less marked than in study 1.

### 3.7. Neurofilament light chain level analysis

#### 3.7.1. Study 1

**Figure 7.**
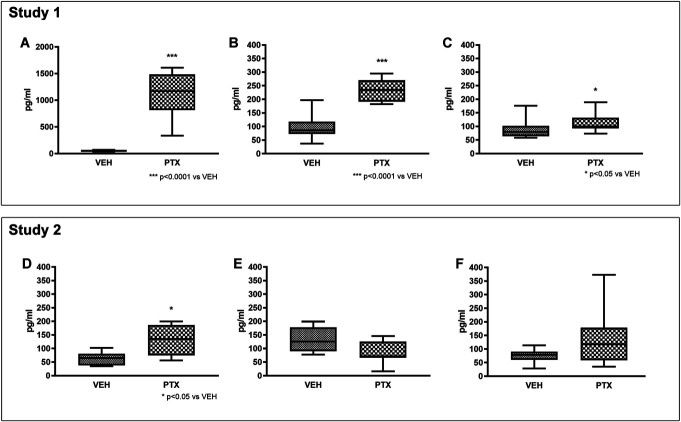
Comparison of neurofilament light (NfL) levels. The NfL levels were measured in study 1 (A = after treatment, B = after 2 weeks of follow-up, C = after 4 weeks of follow-up) and in study 2 (D = after treatment, E = after 2 weeks of follow-up, F = after 4 weeks of follow-up). Paclitaxel (PTX) administration induced a significant increase in NfL levels that was evident at each of the examined time points in study 1, while a significant increase in NfL levels was observed only after treatment in study 2, and its magnitude was remarkably lower if compared with that observed at the same time point in study 1.

At the end of treatment (Fig. 7A), a remarkable and highly significant increase in NfL levels was observed in the PTX group (*P* < 0.0001) and this increase persisted after 2 weeks of follow-up (Fig. 7B) and until the end of the follow-up period (Fig. 7C) (*P* < 0.0001 and *P* < 0.05, respectively).

#### 3.7.2. Study 2

At the end of treatment (Fig. 7D), a milder, but statistically significant increase in NfL levels was observed in the PTX group (*P* < 0.05 vs VEH) also in this study; however, this increase was no longer evident after 2 weeks of follow-up (Fig. 7E) and at the end of the follow-up period (Fig. 7F).

## 4. Discussion

The translation of the animal studies to the clinical level is a very delicate step, partially due to unavoidable species differences but also due to specific technical features of the preclinical models.

Different PIPN models have been developed in mice, with a very wide range of treatment schedule and duration, administered dose, route of administration, assessment methods, and strains (with C57BL/6J appearing to be the most suitable for these studies).^21,29^ Most mouse models of PIPN produce nocifensive behavior following treatment; however, from a translational perspective, it is worth recognizing that pain is predominantly present as an acute symptom in PTX-treated patients. In fact, paresthesias, numbness, loss of tactile perception, and only rarely mild motor weakness are the chronic symptoms responsible for treatment modification in the clinical setting as they may become permanent.^2,20,23,28,39,41^

Therefore, any PIPN model aimed at mimicking the full clinical spectrum occurring in PTX-treated patients must reproduce both painful sensations and overt pathological changes in the peripheral nerves.

To verify if one of the most commonly used PIPN models (study 2)^21,29^ was really able to comply with these requirements, we performed a head-to-head comparison with a well-established PIPN model originally designed on the basis of a typical schedule of PTX in the treatment of breast cancer, ie, using a chronic (4-week) paradigm and a weekly i.v. administration of the drug (study 1).^9^

The assessments performed in study 1 confirmed that PTX administration induces mechanical allodynia, thermal hyperalgesia, reduction in the IENF density, and neurophysiological and pathological signs of a severe distal-to-proximal axonopathy. These features very closely reproduce the typical clinical picture of PTX-treated patients.^1,40^ Moreover, the relatively slow and incomplete recovery of most of these altered parameters in the follow-up period is in agreement with the off-treatment clinical course in a large proportion of affected patients.^28,39^

By contrast, even though in study 2, there was convincing behavioral and pathological evidence of a small fiber neuropathy with rapid and complete recovery in the follow-up, the neurophysiological and pathological analysis of the peripheral nerves failed to demonstrate any alteration.

The measurement of NfL levels, a serological surrogate biomarker that is gaining increasing interest in preclinical experiments, but also in clinical trials,^14,20,25^ confirmed the marked difference existing in the severity of PIPN in the 2 models.

Based on these remarkable differences, it appears that the PIPN model investigated in study 2 is effective in reproducing the acute, painful phase of clinical PIPN, but only the model described in study 1 reliably replicates the complete set of symptoms and instrumental/pathologic changes of clinical PIPN.

The reasons for these differences can be several. First, the single and total doses of PTX are much higher in study 1 than in study 2. This difference is potentially very relevant since it allows the exposure of larger amounts of PTX to the peripheral nervous system in study 1 vs study 2. Second, the models are based on a different route of administration. The i.v. route used in study 1 was primarily selected to mimic the clinical use of the drug. Moreover, in a PIPN rat model, the 4-week-long i.p. administration of the drug induced abdominal bloating, ascites, and, occasionally, death in some animals.^12^ These side effects were never observed in the mice treated with i.p. PTX in study 2, but it is not clear if this absence of local toxicity is due to better species-specific tolerability or the shorter period of treatment in comparison with our original rat study. The different route of administration of PTX has important effects on drug pharmacokinetics and tissue distribution. In fact, using the same dose, the i.v. administration of PTX induces a much higher blood peak concentration (C_max_), while the drug given i.p. remains available at relevant concentrations for a longer period.^37^ We can speculate that these pharmacokinetics differences may be particularly relevant when the tissue distribution of PTX (ie, poorly soluble in water and nearly unable to cross the intact blood–nervous system barrier protecting the brain and most of the course of the peripheral nerves)^13^ is considered. It is possible that accumulation of PTX and direct damage to peripheral nerve axons is more likely with higher blood concentrations achieved with i.v. administration, while accumulation of PTX in the DRG may be more likely at lower, but longer, blood concentrations achieved with i.p. administration due to the greater permeability of blood vessels within the DRG vs peripheral nerves. Involvement of DRG cells might be very relevant in the pathogenesis and onset of different experimental PIPN features. In this study, we did not observe morphological or morphometric changes after PTX exposure in cell soma within the DRG; however, prior studies have reported cellular alterations using immunohistochemical approaches.^30,32,43,44^ For instance, increased expression of activating transcription factor 3 (a marker of cellular injury) was demonstrated in sensory neurons and in a population of satellite cells in the lumbar DRG after PTX administration.^30^ To support the hypothesis that DRG is particularly vulnerable to PTX, and relevant in pain onset, nocifensive behaviors without any pathological evidence of large myelinated fiber damage have been reported using PTX treatment schedules even less intense than the one we tested in study 2.^29^ In addition, the dosing regimen used in study 1 results in more persistent signs of neuropathology weeks after PTX withdrawal allowing for a fairly long window for investigating the off-treatment course of PIPN.

Our experimental observations have an important translational impact not only when animal models are used to generate pathogenic hypotheses but potentially also when their preclinical results are translated into clinical practice or are used to design new clinical trials. In fact, the use of PIPN models that are not able to recapitulate the full spectrum of the clinical disease can be misleading, particularly if pain is not the main target of the investigation. Accordingly, the same bias might be extremely severe in any study designed to prevent or treat PIPN since the absence of a real damage in the peripheral nerves of the mice might prevent reliable translation of the preclinical results to the bedside. It is also worth noting that this model-dependent variability in neurotoxicity is not unique to PTX, and it has already been demonstrated by head-to-head comparison using oxaliplatin, another severely neurotoxic antineoplastic agent,^31^ thus highlighting once more the importance of the proper and informed use of CIPN preclinical in vivo models.

In conclusion, the selection and use of one among the several mice PIPN models should be very carefully weighed according to the real aim of the investigation. It is highly recommended that an extensive assessment including small and large fibers examination at the behavioral, neurophysiological, and pathological levels is performed in any CIPN animal model, to allow a reliable interpretation of the study results, and their safer translation to the clinical setting.

## Conflict of interest statement

The authors have no conflicts of interest to be disclosed in relationship with this study. C.S. and S.A.L. are Italfarmaco S.p.A. employees.
